# Supplementary material for: Identification of photoperiod-induced specific miRNAs in the adrenal glands of Sunite sheep (Ovis aries)
Source: Front Vet Sci. 2022 Jul 22;9:888207. doi: 10.3389/fvets.2022.888207 (PMC9354845; doi:10.3389/fvets.2022.888207)
Supplement: Supplementary file 1 [file Data_Sheet_1.ZIP › Supplementary file/miRDeep2 file.zip!/miRDeep2 file/Know_Structure/LP42A3/oar-mir-539.pdf]

miRBase precursor : oar-mir-539
Total read count : 2107
oar-miR-539-5p read count : 13
oar-miR-539-3p read count : 2094
remaining reads : 0

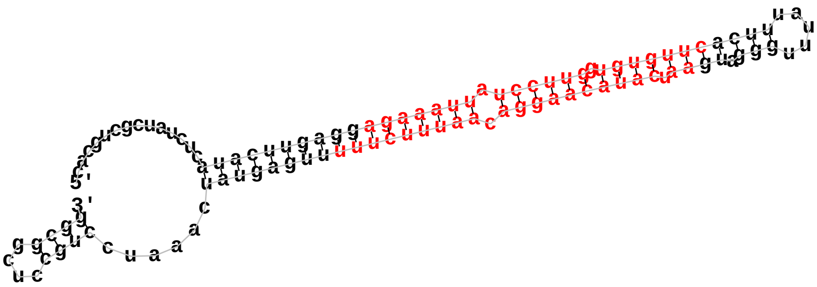

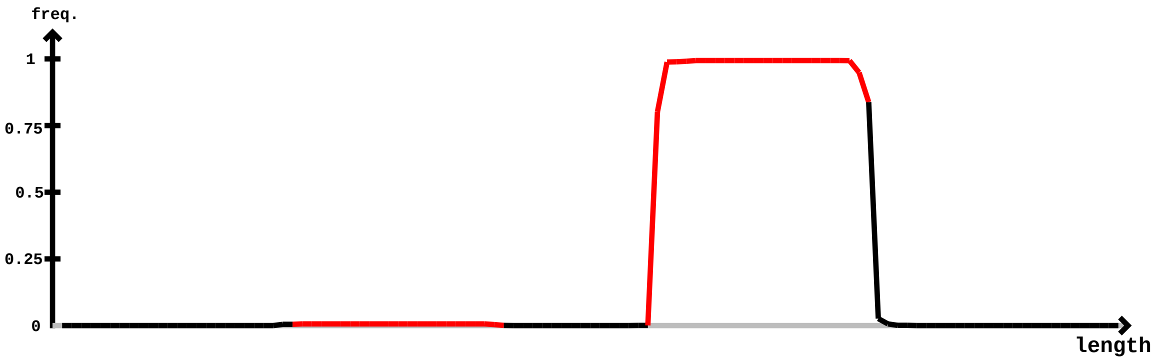

oar-miR-539-3p
oar-miR-539-5p

```
5'- cacgucgcuaucucauacuugaggagaaauuauccuuggugugu ucacuuuauuugggaugaaucauacaaggacaauuucuuuuugaguaucaaauccugccucggcgg -3'   exp
    ..............(((((((((((((((((.((((((.(((((((((((.....))).)))).)))))))))).))))))))))))))))).......((((....))))   reads  mm  sample
    ......................ggagaaauuauccuuggugugu..................................................................   4      0   seq
    ......................ggagaaauuauccuuggugugu u.................................................................   6      0   seq
    ........................agaaauuauccuuggugugu..................................................................   1      0   seq
    ........................agaaauuauccuugguguguu.................................................................   1      0   seq
    ........................agaaauuauccuuggugugu uc................................................................   1      0   seq
    ...........................................................ugaaucauacaaggacaauuuc.............................   1      0   seq
    .............................................................aaucauacaaggacaauuucu............................   93     0   seq
    .............................................................aaucauacaaggacaauuucuu...........................   220    0   seq
    .............................................................aaucauacaaggacaauuucuuu..........................   1359   0   seq
    .............................................................aaucauacaaggacaauuucuuuu.........................   14     0   seq
    .............................................................aaucauacaaggacaauuucuuuuu........................   1      0   seq
    ..............................................................aucauacaaggacaauuucu............................   1      0   seq
    ..............................................................aucauacaaggacaauuucuu...........................   14     0   seq
    ..............................................................aucauacaaggacaauuucuuu..........................   348    0   seq
    ..............................................................aucauacaaggacaauuucuuuu.........................   26     0   seq
    ..............................................................aucauacaaggacaauuucuuuuu........................   5      0   seq
    ...............................................................ucauacaaggacaauuucuuu..........................   2      0   seq
    ................................................................cauacaaggacaauuucuuuu.........................   1      0   seq
    ................................................................cauacaaggacaauuucuuuuu........................   3      0   seq
    .................................................................auacaaggacaauuucuuu..........................   1      0   seq
    .................................................................auacaaggacaauuucuuuu.........................   1      0   seq
    .................................................................auacaaggacaauuucuuuuu........................   2      0   seq
    .................................................................auacaaggacaauuucuuuuuga......................   2      0   seq
```
